# Effects of Sperm DNA Fragmentation on Embryonic Cleavage and Semen Parameters in In Vitro Fertilization Treatment

**DOI:** 10.1155/jare/9920736

**Published:** 2026-02-10

**Authors:** Yu-Li Chuang, Hsiao-Chin Huang, Cheng-Hsuan Wu, Shiao-Hsuan Yang, Yu-Ching Chen, Horng-Der Tsai, Hsin-Hung Wu

**Affiliations:** ^1^ Department of Obstetrics and Gynecology, Changhua Christian Hospital, Changhua, Taiwan, cch.org.tw; ^2^ Nuwa Fertility Center Taichung, Taichung, Taiwan; ^3^ Nuwa Fertility Center Taoyuan, Taoyuan, Taiwan; ^4^ Happy Fertility Center, Changhua, Taiwan; ^5^ Department of Post-Baccalaureate Medicine, College of Medicine, National Chung Hsing University, Taichung, Taiwan, nchu.edu.tw

**Keywords:** embryo, *in vitro* fertilization (IVF), infertility, male infertility, semen analysis, sperm DNA fragmentation (SDF)

## Abstract

**Objective:**

Sperm DNA fragmentation (SDF) may influence embryonic development during in vitro fertilization (IVF) treatment. This study examined the relationship between SDF and embryonic development and correlations between SDF and semen parameters.

**Materials and Methods:**

This retrospective study included couples who received IVF treatment at the Reproductive Medicine Center of Changhua Christian Hospital between July 2020 and May 2022. Couples who experienced repeated implantation failure were suggested to an SDF test, and their most recent IVF treatment results were examined. IVF treatment data were collected for 52 couples. SDF was evaluated using a terminal deoxynucleotidyl transferase‐mediated deoxyuridine triphosphate nick end labeling (TUNEL) assay, calculated as 100 × (number of spermatozoa with fragmented DNA/total number of spermatozoa). Couples were divided into two groups on the basis of their SDF ratio: Group 1 (SDF < 20%) and Group 2 (SDF ≥ 20%). The numbers of embryos at the two‐pronuclear, cleavage, and blastocyst stages were analyzed using a Chi‐square test. Semen parameters were analyzed using a *t* test.

**Results:**

In all IVF treatment cycles, the fertilization rate was significantly lower in Group 2 than in Group 1 (78.62% vs. 68.88%, *p* = 0.0378). The cleavage rate was also significantly lower in Group 2 than in Group 1 (57.89% vs. 37.35%, *p* = 0.0007). The clinical outcome also revealed significantly increased miscarriage rate in Group 2 than in Group 1 (6.25% vs. 14.29%, *p* ≤ 0.005). Subgroup analysis of young mothers revealed lower fertilization and cleavage rates in Group 2, although the differences were not significant. Analysis of semen parameters revealed significantly lower semen concentrations, sperm motility, and progressive motility in Group 2.

**Conclusion:**

SDF affects early embryonic development. A high SDF ratio is significantly associated with low fertilization and cleavage rates and lower semen concentrations, sperm motility, and progressive motility. The embryo derived from higher SDF ratio sperm also has significantly higher miscarriage rate after embryo transfer. In conclusion, SDF analysis can be used as a useful examination for male fertility before assisted reproductive therapy, especially couples with history of repeat IVF treatment.

## 1. Introduction

Semen analysis is a routine examination for male infertility. Recently, sperm DNA fragmentation (SDF) has attracted increasing attention as a potential examination for male infertility [1]. SDF has many underlying causative factors, such as infection [2], advanced age [3], oxygen radicals, endogenous caspases and endonucleases, chemotherapy, radiotherapy, and environmental toxicity [4, 5]. These factors induce chemical changes in DNA during spermatogenesis and lead to DNA damage [4, 5].

A high SDF ratio is an indicator of poor embryonic development [6]. A high SDF ratio also predicts a high miscarriage rate [7] and a low live birth rate in couples receiving in vitro fertilization (IVF) treatment [8]. Therefore, SDF is regarded as a key variable in the context of IVF treatment. Patients who have a history of recurrent pregnancy loss or repeat implantation failure should undergo SDF tests before the procedure [9]. However, there was also opposite opinion in previous study [10]. It is still controversial about the link between IVF treatment result and SDF ratio. SDF data in IVF treatment here in East Asia or Taiwan is deficiency currently. Therefore, we dedicate to figure out the correlation between SDF and embryo development during IVF treatment in our fertility center.

This study collected data from the Fertility Center of Changhua Christian Hospital to determine the effect of SDF on IVF treatment. We examined the correlation between SDF and embryonic development, and established a local repository of related data.

## 2. Materials and Methods

### 2.1. Experimental Design, Patients, and Inclusion and Exclusion Criteria

This retrospective study included couples who received IVF treatment at the Reproductive Medicine Center of Changhua Christian Hospital between July 2020 and May 2022. Couples who experienced repeated implantation failure were suggested an SDF test. The definition of repeat implantation failure is more than 2 times failure of good embryo transfer [11]. Couples whose most recent and not beyond 3 months IVF procedures were examined for embryonic development. Controlled ovarian stimulation followed GnRH antagonist protocol or progestin‐primed ovarian stimulation (PPOS) protocol. Women who were aged older than 44 years at the beginning of IVF treatment were excluded. Women with polycystic ovary syndrome were also excluded. A total of 52 couples were included in the study for analysis.

### 2.2. Semen Analysis and SDF Test

Semen analysis was conducted during each IVF treatment cycle not beyond 3 months around the SDF test. Semen samples were collected after 2–7 days of ejaculatory abstinence. After liquefying, the semen specimen was diluted and pipetted with 50 μL to determine the concentration of sperm. Semen parameters were examined in replicate aliquots of at least 10 μL under a high‐magnification microscope. Semen analyses were conducted by two certified embryologists.

The semen specimens were analyzed for SDF using a terminal deoxynucleotidyl transferase‐mediated deoxyuridine triphosphate nick end labeling (TUNEL) assay. After the samples were completely liquefied, each sample was pipetted into a tube, followed by spinning down for seconds. The specimen was resuspended with pipetting and then diluted with paraformaldehyde to fix for 24 h. After centrifuged, paraformaldehyde was added in the tube, followed by another round of centrifugation. Subsequently, pellets were extracted and resuspended in 1 mL of ice‐cold ethanol (70% vol/vol). This specimen was prepared by TUNEL assay kit (APO‐BrdU TUNEL Assay Kit; Invitrogen; Thermo Fisher Scientific, Inc., USA). The mixture was centrifuged, and the supernatant was discarded, followed by staining solution and rinse buffer. PI/RNase staining buffer was added and allowed to react for 30 min. SDF was analyzed using a confocal microscope (Olympus, Tokyo). The percentage of TUNEL‐positive cells was calculated as follows: 100 × (number of spermatozoa with fragmented DNA/total number of spermatozoa). The test results were retrospectively collected, and the participants were divided into two groups on the basis of their SDF ratio with a threshold of 20%.

### 2.3. IVF Treatment With Intracytoplasmic Sperm Injection (ICSI), and Embryonic Development

All women received IVF procedure and controlled ovarian stimulation followed GnRH antagonist protocol or PPOS protocol. Each woman also received dual stimulation with a GnRH agonist (Lupro, Leuprolide Acetate, Nangkuang Pharmaceutical Co., Ltd., Taiwan) and a human choriogonadotropin (Ovidrel, Choriogonadotropin alfa 250 mg, Merck Serono S.p.A., Italy) at 36 h before ovum pickup.

All oocytes at the MII stage were subjected to an ICSI procedure. Embryos that reached the two‐pronuclear stage (2PN) were maintained in culture. On the third day, each woman was informed of the number of viable embryos. If the number of viable embryos was less than three, these embryos were frozen instead of being maintained in culture until the fifth day.

After fertilization, the numbers of viable embryos at the 2PN, cleavage, and blastocyst stages were documented. Blastocysts were scored using Gardner’s embryo grading system on the fifth day. Viable embryos were defined as those comprising 6–10 cells during the cleavage stage, and blastocyst grade higher than G2BB on the fifth day. Further embryo transfer, including fresh and frozen embryo transfer, and clinical outcomes were also collected. The data were calculated for chemical pregnancy rate, miscarriage rate, and live birth rate in two groups.

### 2.4. Statistical Analysis

Embryonic development was examined using a Chi‐square test, and semen parameters were analyzed using a *t* test. Linear regression was used for the correlation between age and SDF ratio result. Data were analyzed using Microsoft Excel (Microsoft, Redmond, WA, USA), and *p* < 0.05 was considered statistically significant.

## 3. Results

A total of 52 couples were included in this study. Couples were divided into two groups on the basis of SDF ratio. Group 1 (SDF < 20%) comprised 17 couples, and Group 2 (SDF ≥ 20%) comprised 35 couples. Data analysis revealed no significant difference in maternal anti‐Müllerian hormone level, body mass index, or infertility year between the groups. Group 2 had significantly higher maternal and paternal ages compared with Group 1 (Table 1). The mean maternal age in Group 1 was 34.82 years (range: 29–41 years), and the mean maternal age in Group 2 was 36.80 years (range: 30–43 years). The mean paternal age in Group 1 was 34.76 years (range: 28–46 years), and the mean paternal age in Group 2 was 38.60 years (range: 30–53 years). These results indicated a correlation between paternal age and SDF. Compared with Group 1, Group 2 had significantly more IVF procedures and frozen embryo transfer cycles (1.82 vs. 3.17).

**Table 1:** Study group characteristics.

	Group 1 (SDF < 20)	Group 2 (SDF ≥ 20)	*p* value^∗^
	*N* = 17	*N* = 35	
Maternal age (year)	34.82 (29–41)	36.80 (30–43)	0.0282^∗^
Paternal age (year)	34.76 (28–46)	38.60 (30–53)	0.0073^∗^
BMI (kg/m^2^)	22.55	23.52	0.1898
AMH (ng/mL)	2.37	2.41	0.4637
Infertility year	2.53	2.91	0.2714
Past IVF time	1.82	3.17	0.0087^∗^
Infertility cause			
Ovarian dysfunction	8	13	
male factor	3	15	
Advanced maternal age	1	3	
Unexplained	0	5	
Uterine myoma	2	0	
Tubal factor	4	4	
Endometrioma	0	1	
Adenomyosis	1	0	
Genetic problem	0	2	
Hyperprolactinemia	1	0	

^∗^Analyzed using a *t* test, with values below 0.05 indicating a significant difference.

### 3.1. Embryonic Development

Data on embryonic development were collected in all IVF treatment cycles. To determine the progress of fertilization, we conducted a Chi‐square test to analyze the rate of fertilization and the growth rate of each group. In Group 1, 145 MII‐stage oocytes were detected and 114 2PN‐stage oocytes were identified after fertilization, indicating a fertilization rate of 78.62%. On the third day, 66 cleavage‐stage embryos were detected, indicating a cleavage rate of 57.89% (Table 2). In Group 2, 241 MII‐stage oocytes were detected and 166 2PN‐stage oocytes were identified after fertilization, indicating a fertilization rate of 68.88%. On the third day, 62 cleavage‐stage embryos were detected, indicating a cleavage rate of 37.35% (Table 2). Chi‐square analysis revealed that Group 1 had significantly higher fertilization and cleavage rates than Group 2.

**Table 2:** Fertilization results in group 1 (SDF < 20%) and group 2 (SDF ≥ 20%).

	Group 1	Group 2	*p* value^∗^
	(SDF < 20)	(SDF ≥ 20)	
	Patient *N* = 17	Patient *N* = 35	
No. of MII	145	241	
No. of 2PN (Fertilization rate)	114 (78.62%)	166 (68.88%)	0.0378^∗^
No. of Cleavage stage (Cleavage rate)	66 (57.89%)	62 (37.35%)	0.0007^∗^

^∗^Analyzed using a chi‐square test, with values below 0.05 indicating a significant difference.

### 3.2. Couples Who Choose to Maintain Their Embryos in Culture Until the Fifth Day

At our IVF center, couples with a low embryo growth potential or low ovarian reserve may choose to cryopreserve their cleavage‐stage embryos instead of maintaining them in culture until the fifth day. By contrast, couples who have more than three cleavage‐stage embryos typically choose to maintain their embryos in culture until the fifth day. We separately discuss the cases whose embryo was cultured until the fifth day (Table 3). These couples were divided into two groups, namely Group 1 (5 couples, SDF < 20%) and Group 2 (16 couples, SDF ≥ 20%), with fertilization rates of 78.57% and 74.47%, respectively. Group 1 had a higher cleavage rate than did Group 2 (75% vs. 62.85%), although the difference was not significant. By contrast, Group 2 had a higher blastocyst rate on the fifth day than did Group 1 (65.15% vs. 63.63%), although the difference was not significant.

**Table 3:** Results of couples who chose to maintain their embryos in culture until fifth day.

	Group 1	Group 2	*p* value^∗^
	(SDF < 20)	(SDF ≥ 20)	
	Patient *N* = 5	Patient *N* = 16	
No. of MII	56	141	
No. of 2PN (Fertilization rate)	44 (78.57%)	105 (74.47%)	0.5450
No. of Cleavage stage (Cleavage rate)	33 (75%)	66 (62.85%)	0.1521
No. of Blastocyst (Blastocyst rate)	21 (63.63%)	43 (65.15%)	0.8818

^∗^Analyzed using a chi‐square test, with values below 0.05 indicating a significant difference.

### 3.3. The Clinical Outcomes of Embryo Transfer Procedure

Patients might have fresh and frozen embryo transfer, with 3rd‐day cleavage stage embryo or blastocyst. There were 16 patients having embryo transfer in Group 1 (SDF < 20%), and 28 patients having embryo transfer in Group 2 (SDF ≥ 20%). There was 1 patient had no embryo transfer procedure in Group 1 and did 7 patients in Group 2 because of poor fertilization result or poor embryo quality (Table 4). The chemical pregnancy rates were 43.75% in Group 1% and 42.86% in Group 2, without significant differences under chi‐square test analysis. The miscarriage rate was 6.25% in Group 1 (SDF < 20%) and 14.29% in Group 2 (SDF ≥ 20%). Group 2 (SDF ≥ 20%) had significantly higher miscarriage rate. The live birth rate in Group 1 was 37.5%, and the live birth rate in Group 2 was 28.57%, indicating poorer clinical outcome after embryo transfer. However, there was no significant difference between two groups.

**Table 4:** Embryo transfer result in group 1 (SDF < 20%) and group 2 (SDF ≥ 20%).

	Group 1	Group 2	*p* value^∗^
	(SDF < 20)	(SDF ≥ 20)	
	Patient *N* = 16	Patient *N* = 28	
Chemical pregnancy rate	43.75%	42.86%	0.954
Miscarriage rate	6.25%	14.29%	< 0.005^∗^
Live birth rate	37.5%	28.57%	0.541

^∗^Analyzed using a chi‐square test, with values below 0.05 indicating a significant difference.

### 3.4. Subgroup Analysis of Couples Aged Younger Than 38 Years

To determine the effect of age on SDF, we divided the participants into those aged younger than 38 years and those aged 38 years or older. In those aged younger than 38 years, we calculated the number of embryos at different stages after fertilization. These couples were divided into two groups: Group 1 (13 couples, SDF < 20%) and Group 2 (18 couples, SDF ≥ 20%) (Table 5). In Group 1, 119 MII‐stage oocytes were detected and 95 2PN‐stage oocytes were identified after fertilization, indicating a fertilization rate of 79.83%. On the third day, 55 cleavage‐stage embryos were detected, indicating a cleavage rate of 57.89%. In Group 2, 136 MII‐stage oocytes were detected and 94 2PN‐stage oocytes were identified after fertilization, indicating a fertilization rate of 69.12%. On the third day, 53 cleavage‐stage embryos were detected, indicating a cleavage rate of 56.38%. Chi‐square analysis revealed that Group 1 outperformed Group 2, although no statistically significant differences were observed. A similar trend (*p* < 0.1) was observed in the fertilization rates of the two groups.

**Table 5:** Subgroup analysis results of couples aged younger than 38 years.

	Group 1	Group 2	*p* value^∗^
	(SDF < 20)	(SDF ≥ 20)	
	Patient *N* = 13	Patient *N* = 18	
No. of MII	119	136	
No. of 2PN (Fertilization rate)	95 (79.83%)	94 (69.12%)	0.0513^†^
No. of Cleavage stage (Cleavage rate)	55 (57.89%)	53 (56.38%)	0.8337

^∗^Analyzed using a chi‐square test, with values below 0.05 indicating a significant difference.

^†^ *p* values below 0.1 indicate a trend.

### 3.5. Subgroup Analysis of Couples Aged 38 Years or Older

Couples aged 38 years or older were divided into two groups: Group 1 (4 couples, SDF < 20%) and Group 2 (17 couples, SDF ≥ 20%) (Table 6). Analysis was challenging because few couples were included in these groups and blastocyst results were absent. The fertilization rate was 73.08% in Group 1 and 68.57% in Group 2, and the cleavage rate was 57.89% in Group 1 and 56.94% in Group 2. Compared with Group 2, Group 1 had a nonsignificantly higher fertilization rate and cleavage rate.

**Table 6:** Subgroup analysis results of couples aged 38 years or older.

	Group 1	Group 2	*p* value^∗^
	(SDF < 20)	(SDF ≥ 20)	
	Patient *N* = 4	Patient *N* = 17	
No. of MII	26	105	
No. of 2PN (Fertilization rate)	19 (73.08%)	72 (68.57%)	0.6552
No. of Cleavage stage (Cleavage rate)	11 (57.89%)	41 (56.94%)	0.9406

^∗^Analyzed using a chi‐square test, with values below 0.05 indicating a significant difference.

### 3.6. Semen Analysis

After the SDF tests, semen analyses were conducted (Table 7). No differences were noted in ejaculatory abstinence duration or semen volume between the two groups. However, the concentration, motility, and progressive motility of sperm were significantly lower in Group 2 than in Group 1, although the values were within the normal ranges specified by the World Health Organization [12]. Linear regression analysis of the correlation between SDF and paternal age revealed a positive correlation, with an *R* ^2^ of 0.1639.

**Table 7:** Semen analysis results.

Semen analysis	Group 1 (SDF < 20)	Group 2 (SDF ≥ 20)	*p* value
	*N* = 17	*N* = 35	
Abstinence (day)	3.88	3.74	0.3319
Volume (mL)	2.96	3.03	0.4456
Concentration (× 10^6^/mL)	78.24	52.56	0.0207^∗^
Motility (%)	56.73	46.90	0.0239^∗^
Progressing (%)	50.14	40.31	0.0399^∗^

^∗^Analyzed using a chi‐square test, with values below 0.05 indicating a significant difference.

## 4. Discussion

Semen analysis is regarded as a routine clinical examination for couples with infertility. Various male‐related factors affect the outcomes of pregnancy, particularly sperm quality and SDF. SDF has many underlying causative factors, including age [3], environmental toxicity, oxidative stress, radiotherapy, chemotherapy, and systemic disease [1, 4, 5].

Many studies have indicated that a high SDF ratio is typically associated with poor pregnancy outcomes and inadequate embryonic development in couples receiving IVF treatment [4, 6, 8, 13, 14]. Although there was opposite opinion that a high SDF ratio does not adversely affect embryonic development [10], many experts still recommend that couples with infertility should undergo SDF tests before they receive IVF treatment [7, 15], especially in cases of a varicocele, repeated pregnancy loss, or repeated implantation failure [15, 16]. Therefore, we attempted to identify a link between SDF and IVF treatment in this study.

Many techniques are currently available for identifying SDF, including TUNEL [17], the sperm chromatin structure assay [9, 18], and the sperm chromatin dispersion test [19]. Each of these techniques has a different threshold. Considering higher sensitivity, we choose TUNEL for testing SDF in our laboratory. We analyzed SDF under a confocal microscope so as to ensure higher accuracy and less bias between different professional technicians. An SDF threshold of 20% was adopted, consistent with previous studies [13, 18]. The participants were divided into two groups on the basis of their SDF ratio.

**Figure 1:**
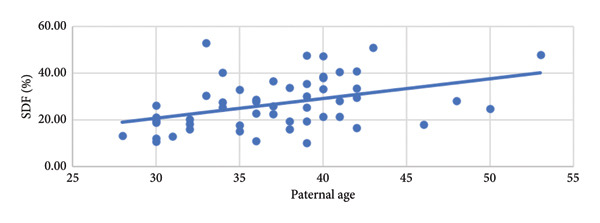
Linear regression analysis results of correlation between SDF and paternal age. *y* = 0.1934*x* + 32.128. *R* ^2^ = 0.1639. *p* = 0.00029^∗^. ^∗^Analyzed using a chi‐square test, with values below 0.05 indicating a significant difference.

Preliminary data analysis (Table 1) revealed a significant difference in paternal age between the two groups, indicating a positive correlation between SDF and overall parental age. The results also indicated a correlation between having a history of IVF treatment failure and having poor IVF treatment outcomes. We also conducted a linear regression analysis to examine the correlation between SDF and paternal age (Figure 1), and our results indicated a significant positive correlation. The result was consistent with previous study and consensus that older patients have a higher SDF ratio [3], particularly in men aged older than 40 years [20–22].

A higher SDF ratio affects the rate of fertilization and the quality of blastocysts in a previous study [13]. It also has a negative effect on the morphokinetic progression of embryos [23]. This study focused on couples who received repeated IVF procedures. The study calculated SDF ratio before couples underwent additional rounds of IVF treatment and discovered that the rates of fertilization and cleavage were lower in Group 2 than in Group 1, consistent with the results of other studies (Table 2) [8, 9].

In our analysis, not all couples chose to maintain their embryos in culture until the fifth day, except embryo number was equal to or above 3. Subgroup analysis included these patients who chose to maintain their embryos in culture until the fifth day. The analysis revealed that the rates of fertilization and cleavage were lower in Group 2 than in Group 1 without significant differences (Table 3). Blastocyst formation rate did not have the same trend between two groups (Table 3), meaning that the number of cases was too small to derive meaningful results. Our findings indicated that embryos derived from better ovarian reserve and oocyte biochemical potential may aid embryo development with repairing the defects from SDF [24].

Reviewing past study, there was evidence that in couples, higher SDF ratio indeed affects pregnancy outcome in IVF treatment [6–8, 13, 14]. We had also collected clinical outcome after embryo transfer, including fresh embryo transfer and frozen embryo transfer. Patients would cancel for the reason of poor embryo quality or no embryo available for embryo transfer procedure. There were 1 patient canceled in Group 1 and 7 patients canceled in Group 2. The cancel rate in Group 2 (SDF ≧ 20%) was 20%, much higher than Group 1 (SDF < 20%), 5.88%. The miscarriage rates were 6.25% in Group 1 and 14.29% in Group 2. The result was significantly higher in Group 2 (SDF ≧ 20%) than in Group 1(SDF < 20%). On the other hand, although without significant differences, the chemical pregnancy rate and live birth rate were both lower in Group 2. The finding indicated that a higher SDF ratio has negative influence on embryo development potential and pregnancy outcome. Our study findings were also consistent with the results of previous studies [6–8, 13, 14].

In the couples with wife aged younger than 38 years, oocytes may play a key role in the repair of DNA defects in embryos [24]. Those aged younger than 38 years were considered to have a higher oocyte quality and embryo growth potential compared with those aged 38 years or older. [24] To determine the effects of maternal age and oocyte quality on embryonic development, we divided the participants on the basis of their maternal age into those aged younger than 38 years and those aged 38 years or older (Tables 5 and 6). In the younger subgroup, the results indicated that the fertilization rate had lower trend in Group 2 than in Group 1 (*p* < 0.1). There was no difference observed in cleavage stage between the two groups, indicating that oocytes from younger age can repair DNA damage in sperm, so that the cleavage rate was similar between two groups (Table 5). As previously proven in IVF treatment, embryo quality with high SDF ratio sperm may be better if the wife is younger [24, 25]. The hypothesis is that young maternal age oocyte has the ability of correction even encounter higher SDF ratio sperm [24, 25]. This finding suggests that both maternal age and paternal factors play a key role in embryonic development in IVF procedure. Although young maternal oocytes may have the capacity to repair sperm DNA damage, this capacity may be limited [24].

Subgroup analysis of women aged 38 years or older (Table 6) revealed that embryos fertilization rate and cleavage rate were lower in Group 2 than in Group 1 without significantly differences. The evidence of embryo development result affected by SDF ratio and maternal age was not evident. These results may have been biased by the small sample size of couples in this subgroup.

In our fertility center, a semen analysis was conducted during each IVF treatment cycle not beyond 3 months around the SDF test (Table 7). Our semen analysis results indicated that the concentration, motility, and progressive motility of sperm were significantly lower in Group 2 than in Group 1, although the mean values of these parameters were within the normal ranges [12]. Exploring the link between SDF and sperm motility, mitochondria may have a role in it. During the SDF assay procedure, we sometimes found the signal appeared lightly at sperm midpiece, instead of sperm head. This finding indicated sperm DNA fragment also consist of mitochondria DNA fraction. Mitochondrial function plays a key role in sperm motility, for it to generate energy for sperm tail movement. Therefore, more sperm DNA defect was found, more impairment of mitochondria was suspected. Consequently, sperm with a higher SDF ratio often exhibit reduced motility and progressive motility [1, 26, 27]. Even men with normozoospermic infertility may have a high SDF ratio [27]. Hence, sperm DNA defects may go undetected if only a conventional semen analysis is conducted before the initiation of assisted reproductive therapy.

In the majority of clinical scenarios, semen analysis is the primary diagnostic tool used during male infertility consultations. Evidence suggests that a high SDF ratio may be present even in men with normozoospermic infertility [27]. In this study, we observed cases in which men had normal semen analysis results but elevated SDF ratios. These findings indicate that solely relying on semen analysis may lead to the omission of infertile men with a high SDF ratio. When men exhibit low sperm motility or progressive motility or present with conditions such as asthenospermia or asthenozoospermia, SDF testing should be also considered before the initiation of IVF treatment to enable timely intervention. In addition, for couples with unexplained infertility and repeat failure in IVF treatment, both semen analysis and SDF testing should be conducted before the initiation of another IVF treatment cycle [15].

This study is limited by its small sample size, which may have introduced bias and reduced the likelihood of observing statistically significant differences. Currently, limited data are available on SDF testing in Taiwan. One of the strengths of this study is focusing on the relationship between SDF ratio and repeated IVF treatment failure couples, indicating a higher SDF ratio might be the cause of the repeated failure in IVF treatment. This study also established evidence that SDF ratio correlating with paternal age. After establishing our laboratory’s SDF ratio threshold, we intend to collect additional clinical data for further analyses.

## 5. Conclusion

In our center, we focused on repeat IVF treatment cases and used the SDF ratios of men for analysis. We discovered that sperm with an SDF ratio of 20% or higher—measured using a TUNEL test—was associated with significantly worse IVF treatment embryonic outcomes, including reduced fertilization and cleavage. However, worse but not significant differences were observed in blastocyst formation. Embryos derived from higher SDF ratio sperm also had lower chemical pregnancy rate and live birth rate. The miscarriage rate also had significantly higher in the higher SDF ratio group. In young women, oocytes repaired DNA defects in sperm, so that the embryo result had no significant differences between two groups. We also discovered that higher SDF ratios were significantly associated with older paternal age, low sperm concentration, motility, and progressive motility, although the values of these parameters were within the normal ranges according to the World Health Organization guideline. Given the effect of IVF treatment on embryonic development and the limitations of interpreting semen analyses alone, SDF testing should be considered a routine examination of infertility couples with repeated IVF treatment failure history.

## Ethics Statement

This study was approved by the institutional review board of Changhua Christian Hospital. (IRB number is 240136).

## Conflicts of Interest

The authors declare no conflicts of interest.

## Author Contributions

Yu‐Li Chuang: data curation, formal analysis, investigation, and writing. Hsiao‐Chin Huang, Shiao‐Hsuan Yang, and Yu‐Ching Chen: methodology and validation. Cheng‐Hsuan Wu: methodology, validation, and resources. Horng‐Der Tsai: conceptualization, methodology, and resources. Hsin‐Hung Wu: conceptualization, supervision, validation, and project administration.

## Funding

No funding was received for this manuscript.

## Data Availability

The data that support the findings of this study are available upon request from the corresponding author.

## References

[1] Agarwal A. , Panner Selvam M. K. , Baskaran S. , and Cho C. L. , Sperm DNA Damage and Its Impact on Male Reproductive Health: A Critical Review for Clinicians, Reproductive Professionals and Researchers, Expert Review of Molecular Diagnostics. (2019) 19, no. 6, 443–457, 10.1080/14737159.2019.1614916, 2-s2.0-85066918393. 31056976

[2] Sergerie M. , Mieusset R. , Croute F. , Daudin M. , and Bujan L. , High Risk of Temporary Alteration of Semen Parameters After Recent Acute Febrile Illness, Fertility and Sterility. (2007) 88, no. 4, 970 e1–970 e7, 10.1016/j.fertnstert.2006.12.045, 2-s2.0-34848890912. 17434502

[3] Moskovtsev S. I. , Willis J. , and Mullen J. B. , Age-Related Decline in Sperm Deoxyribonucleic Acid Integrity in Patients Evaluated for Male Infertility, Fertility and Sterility. (2006) 85, no. 2, 496–499, 10.1016/j.fertnstert.2005.05.075, 2-s2.0-31644438193. 16595239

[4] Sakkas D. and Alvarez J. G. , Sperm DNA Fragmentation: Mechanisms of Origin, Impact on Reproductive Outcome, and Analysis, Fertility and Sterility. (2010) 93, no. 4, 1027–1036, 10.1016/j.fertnstert.2009.10.046, 2-s2.0-77049084953. 20080235

[5] Simon L. , Emery B. R. , and Carrell D. T. , Review: Diagnosis and Impact of Sperm DNA Alterations in Assisted Reproduction, Best Practice and Research Clinical Obstetrics and Gynaecology. (2017) 44, 38–56, 10.1016/j.bpobgyn.2017.07.003, 2-s2.0-85029546628. 28935366

[6] Borges E.Jr., Zanetti B. F. , Setti A. S. , Braga D. P. , Provenza R. R. , and Iaconelli A. , Sperm DNA Fragmentation is Correlated With Poor Embryo Development, Lower Implantation Rate, and Higher Miscarriage Rate in Reproductive Cycles of Non-Male Factor Infertility, Fertility and Sterility. (2019) 112, no. 3, 483–490, 10.1016/j.fertnstert.2019.04.029, 2-s2.0-85066939382. 31200969

[7] Robinson L. , Gallos I. D. , Conner S. J. et al., The Effect of Sperm DNA Fragmentation on Miscarriage Rates: A Systematic Review and Meta-Analysis, Human Reproduction. (2012) 27, no. 10, 2908–2917, 10.1093/humrep/des261, 2-s2.0-84866388353. 22791753

[8] Osman A. , Alsomait H. , Seshadri S. , El-Toukhy T. , and Khalaf Y. , The Effect of Sperm DNA Fragmentation on Live Birth Rate After IVF or ICSI: A Systematic Review and Meta-Analysis, Reproductive BioMedicine Online. (2015) 30, no. 2, 120–127, 10.1016/j.rbmo.2014.10.018, 2-s2.0-84922600191. 25530036

[9] Esteves S. C. , Zini A. , and Coward R. M. , Best Urological Practices on Testing and Management of Infertile Men With Abnormal Sperm DNA Fragmentation Levels: The SFRAG Guidelines, International Brazilian Journal of Urology. (2021) 47, no. 6, 1250–1258, 10.1590/s1677-5538.ibju.2020.1004. 33566471 PMC8486448

[10] Bronet F. , Martinez E. , Gaytan M. et al., Sperm DNA Fragmentation Index Does Not Correlate With the Sperm or Embryo Aneuploidy Rate in Recurrent Miscarriage or Implantation Failure Patients, Human Reproduction. (2012) 27, no. 7, 1922–1929, 10.1093/humrep/des148, 2-s2.0-84863591232. 22537817

[11] Cimadomo D. , de los Santos M. J. , Griesinger G. et al., Eshre Good Practice Recommendations on Recurrent Implantation Failure, Human Reproduction Open. (January 2023) 2023, no. 3, 10.1093/hropen/hoad023. PMC10270320 37332387

[12] WHO , Examination and Processing of Human Semen, 2021, 6th edition, WHO.

[13] Seli E. , Gardner D. K. , Schoolcraft W. B. , Moffatt O. , and Sakkas D. , Extent of Nuclear DNA Damage in Ejaculated Spermatozoa Impacts on Blastocyst Development After *In Vitro* Fertilization, Fertility and Sterility. (2004) 82, no. 2, 378–383, 10.1016/j.fertnstert.2003.12.039, 2-s2.0-4143065747. 15302287

[14] Dar S. , Grover S. A. , Moskovtsev S. I. , Swanson S. , Baratz A. , and Librach C. L. , *In Vitro* Fertilization–Intracytoplasmic Sperm Injection Outcome in Patients With a Markedly High DNA Fragmentation Index (> 50%), Fertility and Sterility. (2013) 100, no. 1, 75–80, 10.1016/j.fertnstert.2013.03.011, 2-s2.0-84879694193. 23562046

[15] Esteves S. C. , Zini A. , Coward R. M. et al., Sperm DNA Fragmentation Testing: Summary Evidence and Clinical Practice Recommendations, Andrologia. (2021) 53, no. 2, 10.1111/and.13874. PMC7988559 33108829

[16] Agarwal A. , Farkouh A. A. , Saleh R. et al., Controversy and Consensus on Indications for Sperm DNA Fragmentation Testing in Male Infertility: A Global Survey, Current Guidelines, and Expert Recommendations, World Journal of Men’s Health. (2023) 41, no. 3, 575–602, 10.5534/wjmh.220282. PMC10307662 37118960

[17] Evenson D. P. , Larson K. L. , and Jost L. K. , Sperm Chromatin Structure Assay: Its Clinical Use for Detecting Sperm DNA Fragmentation in Male Infertility and Comparisons With Other Techniques, Journal of Andrology. (2002) 23, no. 1, 25–43, 10.1002/j.1939-4640.2002.tb02599.x. 11780920

[18] Sergerie M. , Laforest G. , Bujan L. , Bissonnette F. , and Bleau G. , Sperm DNA Fragmentation: Threshold Value in Male Fertility, Human Reproduction. (2005) 20, no. 12, 3446–3451, 10.1093/humrep/dei231, 2-s2.0-28544441896. 16085665

[19] Fernández J. L. , Muriel L. , Rivero M. T. , Goyanes V. , Vazquez R. , and Alvarez J. G. , The Sperm Chromatin Dispersion Test: A Simple Method for the Determination of Sperm DNA Fragmentation, Journal of Andrology. (2013) 24, no. 1, 59–66, 10.1002/j.1939-4640.2003.tb02641.x. 12514084

[20] Kaarouch I. , Bouamoud N. , Madkour A. et al., Paternal Age: Negative Impact on Sperm Genome Decays and IVF Outcomes After 40 Years, Molecular Reproduction and Development. (2018) 85, no. 3, 271–280, 10.1002/mrd.22963, 2-s2.0-85042605565. 29392876

[21] Yang H. , Li G. , Jin H. , Guo Y. , and Sun Y. , The Effect of Sperm DNA Fragmentation Index on Assisted Reproductive Technology Outcomes and Its Relationship With Semen Parameters and Lifestyle, Translational Andrology and Urology. (2019) 8, no. 4, 356–365, 10.21037/tau.2019.06.22, 2-s2.0-85072116615. 31555559 PMC6732090

[22] Das M. , Al-Hathal N. , San-Gabriel M. et al., High Prevalence of Isolated Sperm DNA Damage in Infertile Men With Advanced Paternal Age, Journal of Assisted Reproduction and Genetics. (2013) 30, no. 6, 843–848, 10.1007/s10815-013-0015-0, 2-s2.0-84879844852. 23722935 PMC3696445

[23] Wdowiak A. , Bakalczuk S. , and Bakalczuk G. , The Effect of Sperm DNA Fragmentation on the Dynamics of the Embryonic Development in Intracytoplasmatic Sperm Injection, Reproductive Biology. (2015) 15, no. 2, 94–100, 10.1016/j.repbio.2015.03.003, 2-s2.0-84947040730. 26051457

[24] Martin J. H. , Aitken R. J. , Bromfield E. G. , and Nixon B. , DNA Damage and Repair in the Female Germline: Contributions to ART, Human Reproduction Update. (2019) 25, no. 2, 180–201, 10.1093/humupd/dmy040, 2-s2.0-85062166240. 30541031

[25] Winship A. L. , Stringer J. M. , Liew S. H. , and Hutt K. J. , The Importance of DNA Repair for Maintaining Oocyte Quality in Response to Anti-Cancer Treatments, Environmental Toxins and Maternal Ageing, Human Reproduction Update. (2018) 24, no. 2, 119–134, 10.1093/humupd/dmy002, 2-s2.0-85043313123. 29377997

[26] Maettner R. , Sterzik K. , Isachenko V. et al., Quality of Human Spermatozoa: Relationship Between High-Magnification Sperm Morphology and DNA Integrity, Andrologia. (2014) 46, no. 5, 547–555, 10.1111/and.12114, 2-s2.0-84899646533. 23692628

[27] Belloc S. , Benkhalifa M. , Cohen-Bacrie M. , Dalleac A. , Amar E. , and Zini A. , Sperm Deoxyribonucleic Acid Damage in Normozoospermic Men is Related to Age and Sperm Progressive Motility, Fertility and Sterility. (2014) 101, no. 6, 1588–1593, 10.1016/j.fertnstert.2014.02.006, 2-s2.0-84901813277. 24690240

